# MicroRNA-1 down-regulates proliferation and migration of breast cancer stem cells by inhibiting the Wnt/β-catenin pathway

**DOI:** 10.18632/oncotarget.5873

**Published:** 2015-10-19

**Authors:** Tong Liu, Kebang Hu, Zuowei Zhao, Guanglei Chen, Xunyan Ou, Hao Zhang, Xin Zhang, Xiaofei Wei, Dan Wang, Meizi Cui, Caigang Liu

**Affiliations:** ^1^ Department of Breast Surgery, Harbin Medical University Cancer Hospital, Harbin, China 150000; ^2^ Department of Urology, First Hospital of Jilin University, Changchun, China 130021; ^3^ Department of Breast Cancer, Breast Disease and Reconstruction Center, Breast Cancer Key Lab of Dalian, the Second Hospital of Dalian Medical University, Dalian, China 114006; ^4^ Cancer Center, the First Hospital of Jilin University, Changchun, China 130021

**Keywords:** breast cancer stem cells, miRNA profile

## Abstract

We investigated the miRNA profiles of breast cancer stem cells (CSCs) and non-CSC tumor cells by miRNA microarray and determined the effect of altered miR-1 expression on proliferation and migration of breast CSCs. The potential targets of miR-1 in the Wnt/β-catenin signaling were characterized by bioinformatics analysis and luciferase assay. We found that 14 miRNAs were up-regulated and 13 were down-regulated in the ESA^+^CD44^+^CD24^−^lineage^−^ CSCs, related to ESA^+^CD44^−^CD24^+^lineage^−^ non-CSC tumor cells. The miR-1 expression was associated inversely with aggressiveness of breast cancers. Furthermore, enhanced miR-1 expression decreased the percentages of SKBR3/CSCs and miR-1 inhibition increased the percentages of MCF-7/CSCs. Enhanced miR-1 expression significantly reduced the Frizzled 7 and Tankyrase-2 (TNKS2)-regulated luciferase activity in 293T cells and decreased Frizzled 7, TNKS2, c-Myc, octamer-binding transcription factor 4 (Oct4) and Nanog expression and the ratios of nuclear to cytoplasmic β-catenin as well as β-catenin-dependent luciferase activity in breast CSCs *in vitro*. miR-1 inhibited proliferation, migration and wound healing of breast CSCs *in vitro*. Enhanced miR-1 expression inhibited the growth of implanted MCF-7/CSCs while miR-1 inhibition promoted the growth of implanted MCF-7/CSCs *in vivo*. Our data indicate that miR-1 down-regulates breast CSC stemness, proliferation and migration by targeting the Frizzled 7 and TNKS2 to inhibit the Wnt/β-catenin signaling.

## INTRODUCTION

Cancer stem cells (CSCs) are associated with the recurrence, metastasis and drug resistance of cancer [1, 2]. Our previous studies and those of others have shown that ESA^+^/CD44^+^/CD24^−^/lin^−^ breast CSCs express stemness-related transcription factors of octamer-binding transcription factor 4 (Oct4), Nanog and sex determining region Y-box 2 (Sox2) as well as mesenchymal features of N-cadherin, vimentin and Zinc finger protein SNAI1 (Snail) [3–5]. The levels of Oct4 and Nanog expression in breast cancer tissues are positively correlated with poor survival of patients with triple negative breast cancer [6]. In addition, aberrant activation of the Wnt/β-catenin signaling is associated with the stemness of breast CSCs [7, 8]. The Wnt binds to its co-receptors of Frizzled to activate Dsh, which recruits glycogen synthase kinase-3 beta (GSK-3β), promoting β-catenin nuclear translocation and downstream gene expression, such as c-Myc. Moreover, the Wnt/β-catenin signaling is enhanced by Tankyrase (TNKS), which promotes the degradation of major inhibitor of Axin. Hence, down-regulating the Wnt/β-catenin signaling may be critical for inhibition of the stemness and activity of breast CSCs. However, little is known about natural inhibitor of the Wnt/β-catenin signaling in breast CSCs.

miRNA can bind to the 3′ terminal untranslation region (UTR) of targeted mRNAs to inhibit their translation and promote their degradation. miRNAs can act either as a oncogenic factor or suppressor during the development and progression of breast cancer. Previous studies have shown that some miRNAs are highly expressed in tumor tissues while others are down-regulated in tumor tissues [9–11]. However, little is known about miRNA expression profile in breast CSCs and which miRNAs inhibit the Wnt/β-catenin signaling and the stemness and activity of breast CSCs.

In the present study, we determined the profile of the Wnt/β-catenin signal-related miRNA expression in ESA^+^CD44^+^CD24^−^lin^−^ CSCs and ESA^+^CD44^−^CD24^+^lin^−^ non-CSC tumor cells from six surgical breast cancer tissues by miRNA microarray and characterized the levels of miR-1 expression in different types of breast cancers and serum samples by quantitative RT-PCR. Furthermore, we characterized the potential targets of miR-1 in the Wnt/β-catenin signaling by bioinformatics analysis and luciferase assay, and tested the impact of altered miR-1 expression on proliferation, migration and wound healing of breast CSCs *in vitro*. Our data indicated that miR-1 bound to the Frizzled 7 and TNKS2 to inhibit the Wnt/β-catenin signaling, stemness, proliferation and migration of breast CSCs.

## RESULTS

### miR-1 expression is down-regulated in breast CSC and is associated inversely with aggressiveness of breast cancer

**Figure 1:**
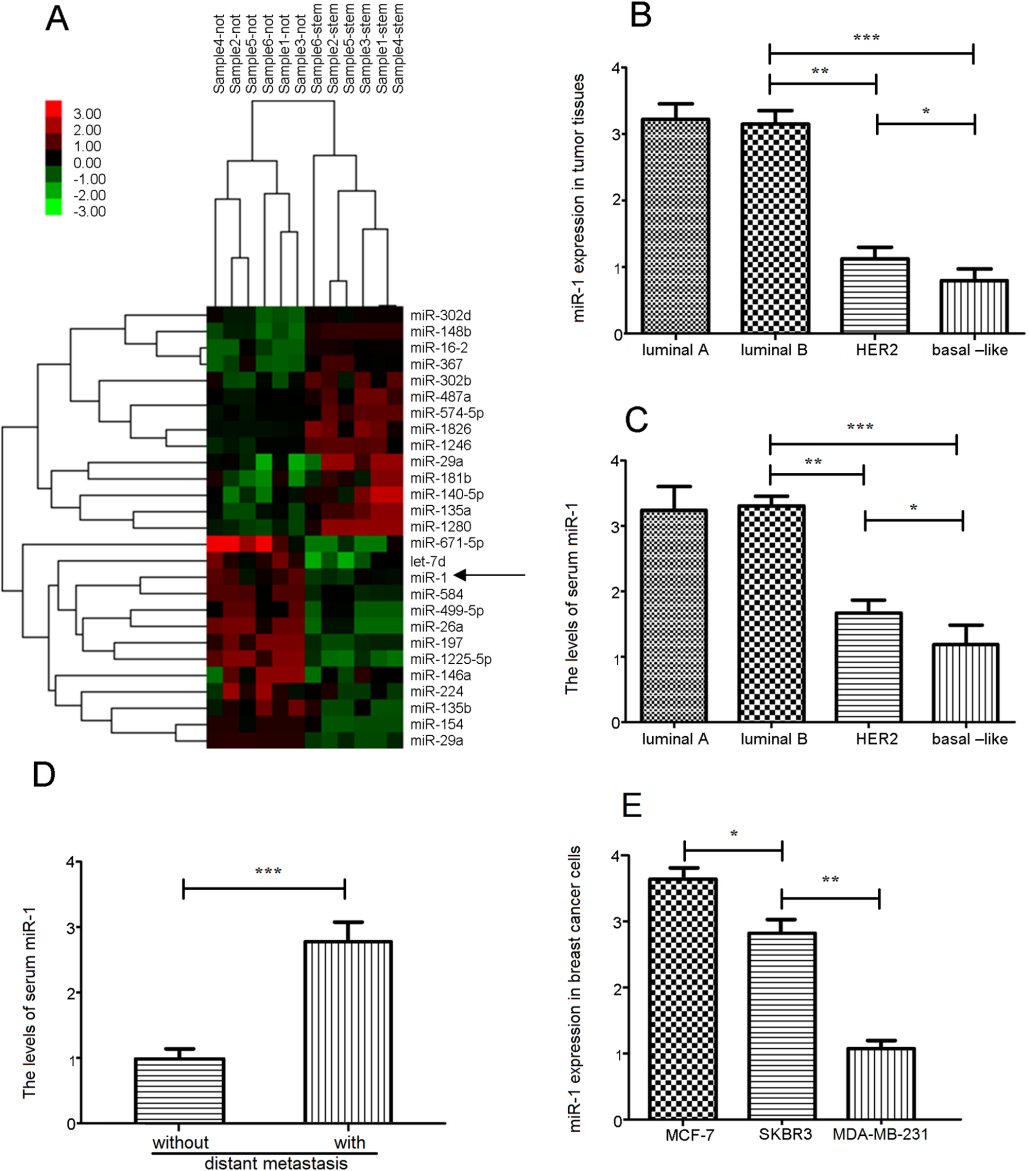
The expression of miRNAs in breast cancer **A.** The miRNA expression profile in six paired of breast CSC and non-CSC samples was characterized by miRNA microarray. The relative levels of miR-1 expression in different types of breast cancer tissue **B.** and serum samples **C.** were determined by quantitative RT-PCR. **D.** The relative levels of serum miRNA in patients (*n* = 18) with or without lymph node metastasis (*n* = 27). **E.** The levels of miR-1 expression in different types of breast cancer cell lines. Data are representative profile of clustered miRNA in individual samples and expressed as the mean ± SD of each group of samples from at least three separate experiments. *N* = 20 for luminal A; 10 for luminal B; 5 for Her2+; 10 for basal-like. *p < 0.05; **p < 0.01; ***p < 0.001.

To understand the regulatory roles of miRNAs in the function of breast CSC, ESA^+^CD44^+^CD24^−^lin^−^ CSC and ESA^+^CD44^−^CD24^+^lin^−^ non-CSC tumor cells were sorted from six freshly surgical breast cancer tissue samples and the profile of the Wnt/β-catenin signal-related miRNA expression in these cells were characterized by miRNA microarray (Figure 1A). In comparison with ESA^+^CD44^−^CD24^+^lin^−^ non-CSC tumor cells, 14 miRNAs were up-regulated and 13 were down-regulated in the ESA^+^CD44^+^CD24^−^lin^−^ CSC cells (Supplementary Table S2). Quantitative RT-PCR analysis of 45 breast cancer tissues and their corresponding serum samples indicated the levels of miR-1 expression in the Her2+ tumors were significantly lower than that in the luminal A and B tumors, but significantly higher than that in the basal-like tumors (Figure 1B). A similar pattern of relative levels of serum miR-1 was detected in those patients with different types of breast cancer (Figure 1C). Furthermore, the levels of serum miR-1 in those with lymph node metastatic tumors were significantly higher than that in those without lymph node metastasis (Figure 1D). Moreover, the levels of miR-1 in SKBR3 were significantly lower than that in MCF-7, but significantly higher than that in the MDA-MB-231 cells (Figure 1E). Together, miR-1 expression was down-regulated in breast CSCs and associated inversely with the aggressiveness of breast cancer in this population.

### miR-1 over-expression reduces the percentages of breast CSCs in breast cancer cell lines

**Figure 2:**
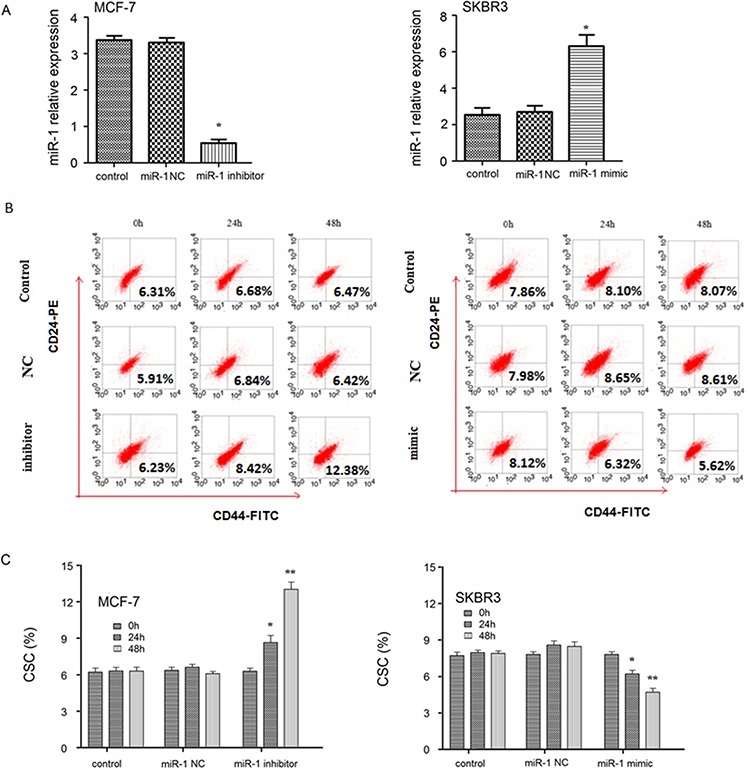
Altered miR-1 expression changes the percentages of breast CSC in breast cancer cell lines MCF-7 and SKBR3 cells were transfected with, or without, miR-1 NC, miR-1 inhibitor, or miR-1 mimic for 24 or 48 h. The relative levels of miR-1 expression in different groups of cells were determined by quantitative RT-PCR **A.** The frequency of CD44+CD24− CSCs was determined by flow cytometry **B–C.** Data are representative FACS charts and expressed as the mean ± SD of each group of cells from three separate experiments. *p < 0.05, **p < 0.01 vs. the control CSCs.

To understand the role of miR-1 in the function of breast CSCs, MCF-7 and SKBR3 cells were transfected with, or without, miR-1NC, miR-1 mimic or miR-1inhibitor for 24–48 h to obtain control MCF-7/CSC, MCF-7/miR-1NC, MCF-7/miR-1 inhibitor, control SKBR3, SKBR3/miR-1NC, and SKBR3/miR-1mimic cells, respectively. RT-PCT indicated that transfection with miR-1 inhibitor significantly reduced miR-1 expression in MCF-7/miR-1 inhibitor cells while transfection with miR-1 mimic significantly elevated miR-1 expression in SKBR3/miR-1 mimic cells (Figure 2A). Flow cytometry analysis revealed that there was no significant difference in the percentages of CD44+CD24− CSCs between the control and MCF-7/miR-1NC cells (Figure 2B). In contrast, the percentages of CD44+CD24− CSCs in the MCF-7/miR-1 inhibitor cells significantly increased with time while the percentages of CD44+CD24− CSC in the SKBR3/miR-1mimic cells significantly decreased with time, related to the controls (Figure 2B and 2C). These data indicated that induction of miR-1 over-expression inhibited SKBR3/CSC proliferation while miR-1 silencing enhanced MCF-7/CSC proliferation *in vitro*.

### miR-1 inhibits the Wnt/β-catenin signaling and reduces the stemness of breast CSCs

**Figure 3:**
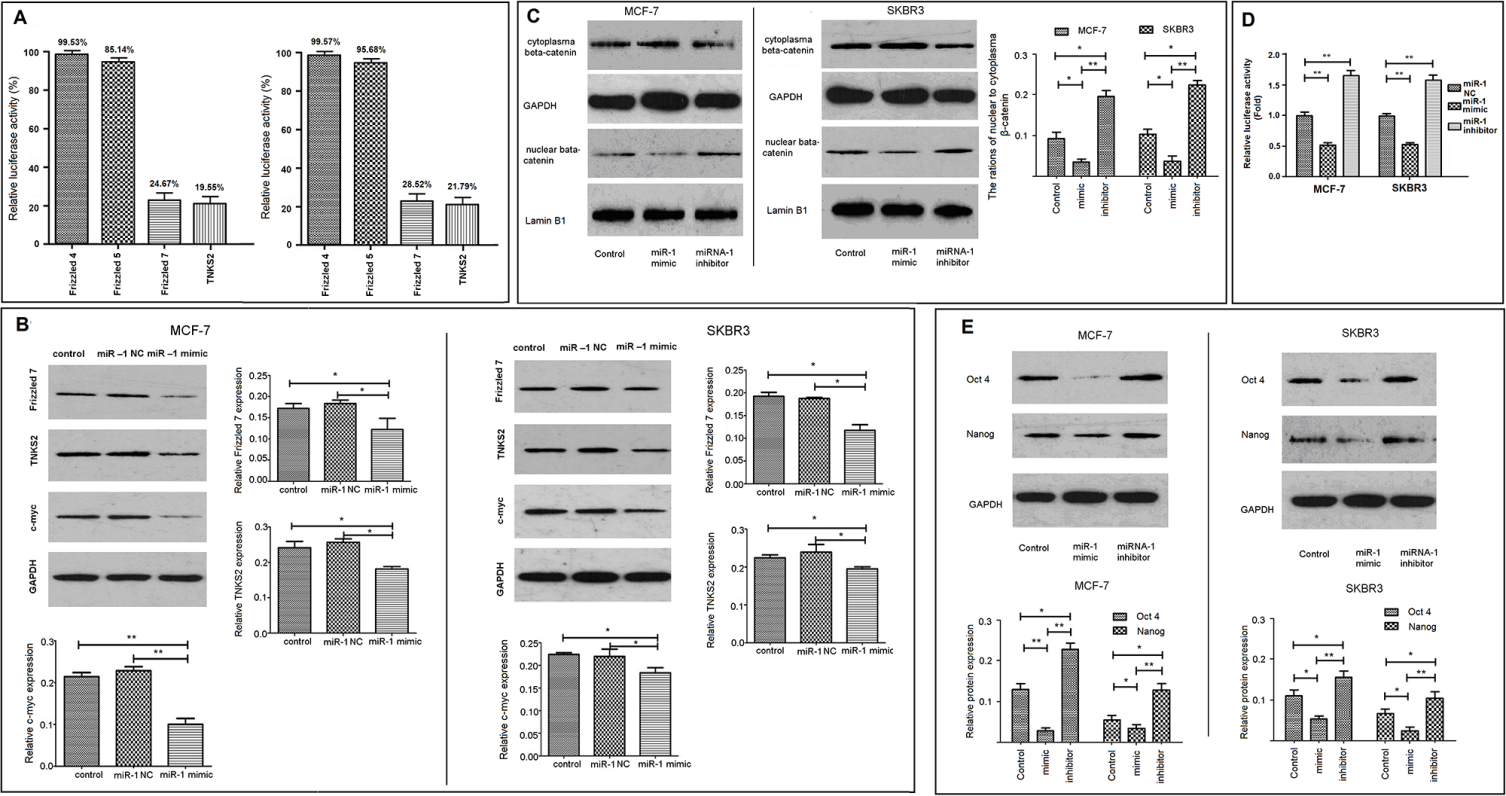
miR-1 inhibits the stemness of breast CSCs by targeting the Wnt/β-catenin signaling 293T cells were transfected with luciferase reporter plasmids containing the 3UTR sequences of the fizzled 4, 5, 7, or TNKS2 and miR-1 or control miR-1NC and the effect of miR-1 on luciferase activity was determined **A.** The luciferase activity in the control cells that had been transfected with miR-1NC were considered as 100%. CD44+CD24− CSC from MCF-7 and SKBR3 cells were sorted and transfected with, or without, miR-1NC, miR-1inhibitor, or miR-1mimic for 24 or 48 h. The relative levels of fizzled 7, TNKS2 and c-Myc to control GAPDH were determined by Western blot **B.** In addition, the relative levels of cytoplasmic and nuclear β-catenin to GAPDH or lumen B1 in each group of cells were determined by Western blot **C.** Moreover, MCF-7/CSC and SKBR3/CSC were transfected with miR-1mimic, miR-1NC or miR-1inhibitor for 24 h. The cells in each group were transfected with TopFlash and pRL-TK for 48 h. The relative levels of β-catenin-dependent firefly luciferase activity in different groups of CSCs were determined by the dual luciferase assay **D.** The relative levels of β-catenin-dependent luciferase activity in the control cells that had been transfected with miR-1NC, TopFlash and pRL-TK were designated as 1. Finally, the relative levels of Oct4 and Nanog expression in each group of cells were determined by Western blot **E.** Data are representative images and expressed as the mean ± SD of each group of cells from three separate experiments. *p < 0.05; **p < 0.01.

Our studies and those of others have shown that aberrant activation of the Wnt/β-catenin signaling promotes proliferation and stemness of CSCs [7, 8]. We found that the 3′UTR of Frizzled 4, Frizzled 5, Frizzled 7, and TNKS2 contained the complementary sequences of miR-1 (Supplementary Table S1). Furthermore, dual-luciferase assay indicated that transfection with miR-1mimic significantly inhibited the levels of Frizzled 7 and TNKS2, but not Frizzled 4 and Frizzled 5, regulated luciferase activity in 293T cells (Figure 3A). Western blot analyses revealed that the relative levels of Frizzled 7, TNKS2 and c-Myc expression in MCF-7/miR-1mimic or SKBR3/miR-1mimic CSCs were significantly lower than that in MCF-7/miR-1NC or SKBR3/miR-1NC CSCs (Figure 3B). In addition, the ratios of nuclear to cytosol β-catenin in MCF-7/miR-1mimic or SKBR3/mimic CSCs were significantly lower than that in the control CSCs while the ratios of nuclear to cytosol β-catenin in MCF-7/miR-1 inhibitor or SKBR3/miR-1 inhibitor CSCs were significantly higher than that in the controls (Figure 3C). Further luciferase assays revealed that enhanced miR-1 expression significantly decreased the relative levels of β-catenin-dependent luciferase activity in MCF-7/miR-1mimic and SKBR3/miR-1mimic CSCs while down-regulated miR-1 expression significantly increased the relative levels of β-catenin-dependent luciferase activity in MCF-7/miR-1inhibitor and SKBR3/miR-1inhibitor CSCs (Figure 3D). Moreover, a similar pattern of Oct4 and Nanog expression was detected in the different groups of CSCs (Figure 3E). Collectively, these data suggest that miR-1 may inhibit the Wnt/β-catenin signaling by reducing the Frizzled 7 and TNKS2 expression in breast CSCs.

### miR-1 inhibits breast CSC and non-CSC tumor cell proliferation

**Figure 4:**
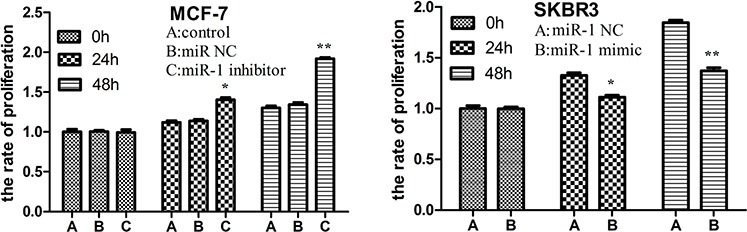
miR-1 inhibits breast CSC proliferation *in vitro* CD44+CD24− CSCs from MCF-7 and SKBR3 cells were transfected with, or without, miR-1 NC, miR-1 inhibitor, or miR-1 mimic for 24 or 48 h. Their proliferation was determined by MTT assay. Data are expressed as the mean rates ± SD of each group of cells from three separate experiments and proliferation of untransfected control CSCs was designated as 1. *p < 0.05; **p < 0.01 vs. the miR-1 NC and control CSCs.

To further characterize the inhibitory effect of miR-1, CD44+CD24− CSC and CD44− CD24+ non-CSC tumor cells were sorted from MCF-7 and SKBR3 cells. We found that relative levels of miR-1 in the non-CSC tumor cells were significantly higher than that in the corresponding CSCs (Supplementary Figure S2A). Following transfection of these cells with, or without, miR-1NC, miR-1inhibitor or miR-1mimic, the rates of MCF-7/CSC, MCF-7/miR-1NC, MCF-7/miR-1inhibitor, SKBR3/miR-1NC and SKBR3/miR-1mimic CSC and non-CSC tumor cell proliferation were determined by MTT. After 24-h or 48-h culture, the proliferation rates of MCF-7/miR-1 inhibitor CSCs were significantly greater than that of the MCF-7/CSC, MCF-7/miR-1NC CSCs (Figure 4). In contrast, the proliferation rates of SKBR3/miR-1mimic CSCs were significantly less than that of the SKBR3/miR-1NC CSCs (Figure 4). A similar pattern of proliferation rates was detected in the different groups of non-CSC breast cancer cells (Supplementary Figure S2B). Hence, miR-1 inhibited breast CSC and non-CSC tumor cell proliferation *in vitro*.

### miR-1 inhibits the migration of breast CSCs

**Figure 5:**
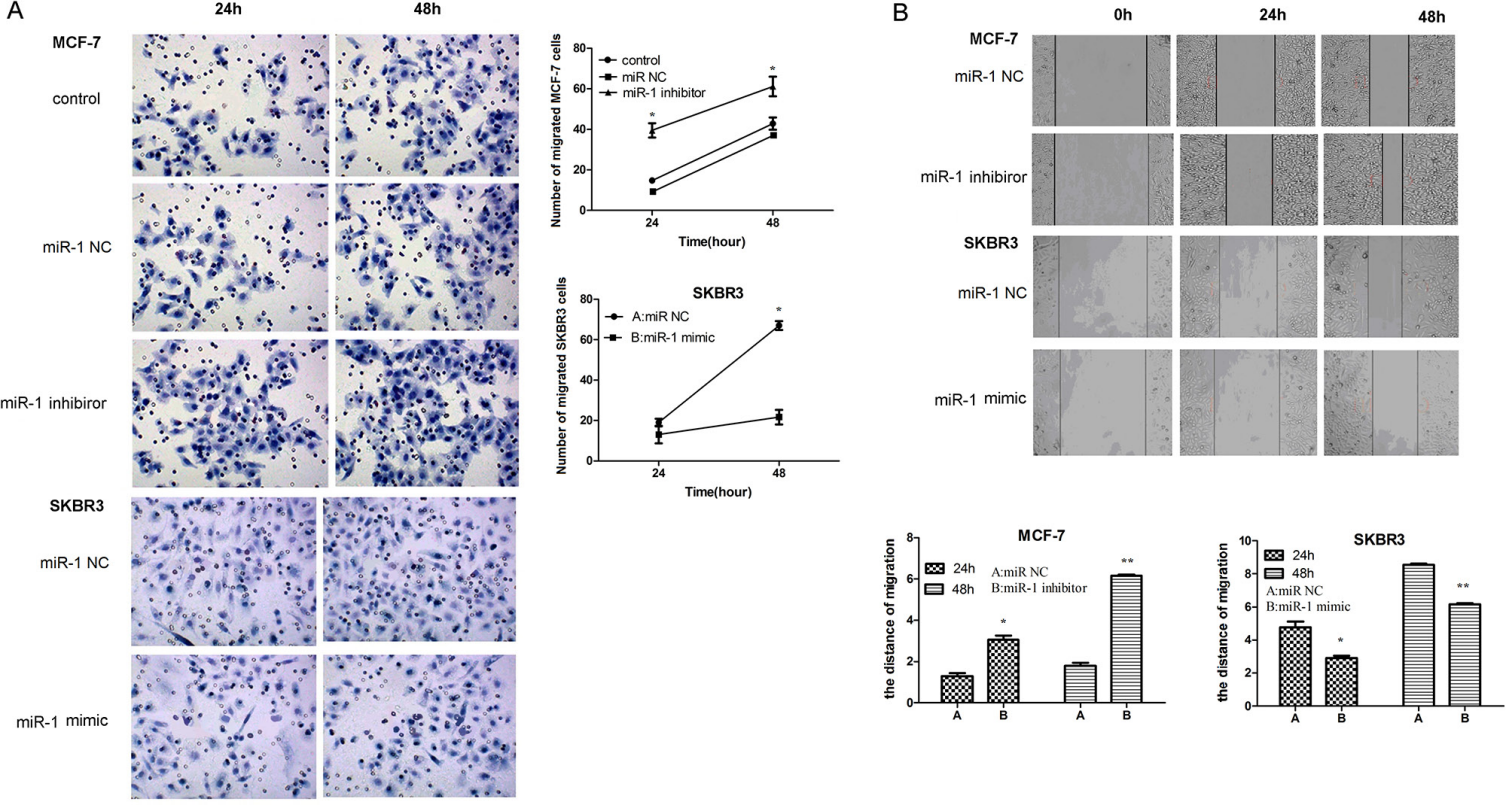
miR-1 inhibits the migration and wound healing of CSC *in vitro* CD44+CD24− CSC from MCF-7 and SKBR3 cells were transfected with, or without, miR-1 NC, miR-1 inhibitor, or miR-1 mimic. Four hours after transfection, the migration of different groups of CSC for the indicated time points was determined by transwell migration assay **A.** In addition, two days after transfection, the wound healing of different groups of cells within 24 or 48 h was tested **B.** Data are representative images and expressed as the mean ± SD of each group of cells from three separate experiments. The number of migrated SKBR3/CSCs was similar to those of SKBR3/miR-1NC CSCs and the migration distance of control MCF-7CSC and SKBR3/CSC was similar to those of MCF-7/miR-1NC and SKBR3/miR-1NC CSCs, respectively (data not shown). *p < 0.05; **p < 0.01 vs. the miR-1 NC-transfected CSCs.

Next, we tested the impact of altered miR-1 expression on the migration of breast CSCs by transwell migration and wound healing assays. First, there was no significant difference in the numbers of migrated cells between the MCF-7/CSCs and MCF-7/miR-1NC CSCs (Figure 5A). Furthermore, the numbers of migrated MCF-7/miR-1 inhibitor CSCs increased with time but the numbers of migrated SKBR3/miR-1mimic CSCs decreased, related to the SKBR3/miR-1NC CSCs. A similar pattern of MCF-7 and SKBR3 CSC migration was observed in wound healing assay (Figure 5B). Together, these data clearly indicated that miR-1 inhibited the migration of breast CSC *in vitro*.

### miR-1 inhibits the growth of implanted breast CSCs *in vivo*

**Figure 6:**
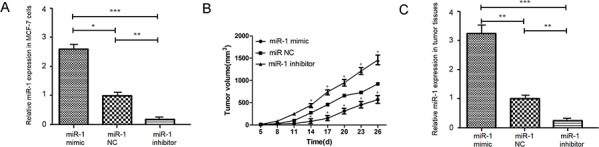
miR-1 inhibits the growth of implanted breast tumors *in vivo* The relative levels of miR-1 expression in the MCF-7/miR-1mimic, MCF-7/miR-1inhibitor or control MCF-7/miR-1NC CSCs were determined by quantitative RT-PCR **A.** The development and growth of implanted CSC-related tumors were monitored at the indicated time points post inoculation **B.** At the end of this experiment (on day 26 post inoculation), the relative levels of miR-1 expression in the different groups of dissected tumors were determined **C.** Data are expressed as the mean ± SD of each group of mice (*n* = 8 per group). *p < 0.05; **p < 0.01; ***p < 0.001 vs. the tumors induced by the MCF-7/miR-1NC CSCs.

Finally, we tested the impact of altered miR-1 expression on the growth of implanted MCF-7 CSCs in mice. After generating MCF-7/miR-1mimic and MCF-7/miR-1inhibitor CSC lines *in vitro*, we found the relative levels of miR-1 expression in the MCF-7/miR-1mimic CSCs were significantly higher than that in the MCF-7/miR-1NC and MCF-7/miR-1inhibitor CSCs (Figure 6A). The relative levels of miR-1 in the MCF-7/miR-1inhbitor CSCs were significantly lower than that in the MCF-7/miR-1NC CSCs. Following implanted with individual types of CSCs, we monitored the growth of implanted tumors and found that the MCF-7/miR-1inhibitor, MCF-7/miR-1mimic, and MCF-7/miR-1NC groups of tumors continually grew (Figure 6B). The volumes of control MCF-7/miR-1NC tumors were significantly smaller than that of MCF-7/miR-1inhibitor tumors, but significantly larger than that of the MCF-7/miR-1mimic tumors (Figure 6B). Further analysis indicated that the relative levels of miR-1 expression in the control MCF-7/miR-1NC CSC tumors were significantly lower than that in the MCF-7/miR-1mimic, but higher than that in the MCF-7/miR-1inhibitor tumors (Figure 6C). These data indicated that miR-1 inhibited the growth of CSC-induced breast cancers *in vivo*.

## DISCUSSION

In this study, we first explored the profiles of the Wnt/β-catenin signal-related miRNA expression in breast CSCs by microarray. We found that 14 miRNAs were up-regulated while 13 miRNAs were down-regulated in breast CSCs, related to that in the non-CSC tumor cells. Our data were different from previous reports [16–18]. The disparity may stem from the different types of cells for microarray. Previous studies have shown that Let-7, miR-200, and miR-30 family, miR-128, miR-34c, miR-16 and miR-205 expression are down-regulated in breast CSCs and they can inhibit the stemness, self-renewal, differentiation and EMT process of breast CSCs by targeting the H-RAS, HMGA2, Bmi-1, Ubc9, ZEB1/2, PRC1/2, Notch4, and Wip1, respectively [19]. In contrast, miR-181, miR-495, and miR-22 expression are up-regulated in breast CSCs and can promote the function of breast CSCs by targeting ATM, REDD1 and TET, respectively [20]. Our data extended previous findings and suggest that imbalance of miRNA expression may be crucial for the maintenance of breast CSC during the development and progression of breast cancer. Given that miR-1 is an important tumor suppressor in other types of cancers and a valuable marker for prognosis of breast cancer we validated the levels of miR-1 expression in breast cancer tissues and serum samples from patients with different types of breast cancers by quantitative RT-PCR. We found that the levels of miR-1 in serum samples and different types of breast cancer tissues were associated negatively with the aggressiveness of different types of breast cancers in this population. The significantly reduced miR-1 expression in patients with aggressive cell-type of breast cancer support the notion that miR-1 is a tumor suppressor of breast cancers [9, 21–22]. Interestingly, we detected significantly higher levels of serum miR-1 in patients with lymph node metastatic breast cancer than those without lymph node metastasis. Because miR-1 is not only expressed by breast cancer cells, but also other types of cells. It is possible that early metastatic breast cancer cells may stimulate other types of cells, such as bone marrow cells, to produce high levels of miR-1. We are interested in further validating the miR-1 expression in breast CSCs and exploring its value in the prognosis of breast cancer.

Our previous studies and those of others have shown that aberrant activation of the Wnt/β-catenin signaling is crucial for the stemness and activity of breast CSCs [23–25]. In this study, we found that miR-1 might bind to the 3′UTR of Fizzled 4, 5, 7 and TNSK2 and transfection with miR-1 mimic dramatically reduced the luciferase activity regulated by the 3′UTR of Fizzled 7 and TNSK2, but not Fizzled 4 and 5 in 293T cells. Furthermore, enhanced miR-1 over-expression significantly down-regulated the Fizzled 7, TSKN2, and c-Myc expression, and reduced the relative ratios of nuclear to cytosol β-catenin in breast CSCs. In contrast, down-regulated miR-1 expression up-regulated the Fizzled 7, TSKN2, and c-Myc expression and increased the relative ratios of nuclear to cytosol β-catenin in breast CSCs. In addition, enhanced miR-1 expression significantly decreased the levels of β-catenin-dependent luciferase activity in MCF-7/miR-1mimic and SKBR3/miR-1mimic CSCs while down-regulated miR-1 expression significantly increased the levels of β-catenin-dependent luciferase activity in MCF-7/miR-1inhibitor and SKBR3/miR-1inhibitor CSCs. These independent evidence clearly indicated that miR-1 inhibited the Fizzled 7 and TSKN2 expression and the Wnt/β-catenin signaling in breast CSCs [26]. miR-1 can target the MET, Slug and PI3KCA to inhibit proliferation and metastasis of different types of tumor cells [27, 28]. To the best of our knowledge, our findings provided the first evidence to demonstrate that miR-1 down-regulates the up-stream Wnt co-receptor and regulator expression to inhibit the Wnt/β-catenin signaling in breast CSCs. Hence, miR-1 may be a natural inhibitor of the Wnt/β-catenin signaling and valuable for intervention of breast cancer.

There are several miRNAs that inhibit or enhance the Wnt/β-catenin signaling during the development of different types of tumor, including breast cancer [27]. miR-135 and miR-142 inhibit the adenomatous polyposis coli (APC) expression to enhance the Wnt/β-catenin signaling and tumor cell proliferation and migration [23, 29] while miR-200a, miR-203, miR-214, miR-1826, miR-320 and others attenuate the Wnt/β-catenin signaling by targeting β-catenin to inhibit tumor cell proliferation and migration [30–36]. Hence, these miRNAs form a complex network to regulate the Wnt/β-catenin signaling during the development and progression of tumors. We are interested in further investigating the network of different miRNAs in regulating the tumorigenesis of breast CSCs.

Inhibition of the Wnt/β-catenin signaling can attenuate proliferation and epithelial mesenchymal transition (EMT) process of breast CSCs [37, 38]. Our previous study has shown that Nestin promotes the survival, proliferation and invasion of breast CSCs by enhancing the Oct4 and Nanog expression [8] and a recent study indicates that Pterostilbene modulates the NF-kB/miR488 circuit to inhibit M2 macrophage-regulated breast CSC proliferation and migration *in vitro* [39]. In this study, we found that enhanced miR-1 expression significantly inhibited proliferation, migration and wound healing of breast CSCs while miR-1 inhibition significantly enhanced proliferation, migration and wound healing of breast CSCs *in vitro*. Furthermore, enhanced miR-1 also inhibited the proliferation of non-CSC tumor cells while miR-1 inhibition significantly increased the proliferation rates of non-CSC tumor cells *in vitro*. Therefore, miR-1 not only inhibited proliferation of breast CSC, but also non-CSC tumor cells *in vitro*. It is important to continually investigate how miR-1 inhibits breast non-CSC tumor cell proliferation. Moreover, enhanced miR-1 stable expression inhibited the growth of implanted breast CSC-related tumors while miR-1 inhibition promoted the growth of implanted breast CSC-related tumors *in vivo*. In addition, miR-1 inhibition enhanced the relative levels of Oct4 and Nanog expression in breast CSCs while enhanced miR-1 expression attenuated the relative levels of Oct4 and Nanog expression in breast CSCs. Our data extended previous findings [17, 30] and demonstrate that miR-1 attenuates the stemness, proliferation and migration of breast CSCs. Given that breast CSCs are crucial for the recurrence and metastasis of breast cancer, miR-1-based therapies may be promising in prevention and intervention of breast cancer recurrence and metastasis.

In summary, our data indicated differential expression of miRNAs between breast CSCs and non-CSC tumor cells and miR-1 expression was associated negatively with the aggressiveness of breast cancers. miR-1 inhibited the stemness, proliferation, migration and wound healing of breast CSCs, associated with binding to the Fizzled 7 and TNSK2 to inhibit the Wnt/β-catenin signaling. Enhanced miR-1 expression inhibited the growth of implanted breast CSC-related tumors *in vivo*. Our data may provide new insights into regulating the stemness, self-renewal and migration of breast CSCs. Our novel findings may aid in design of new therapies for intervention of breast cancer.

## MATERIALS AND METHODS

### Subjects

A total of 45 patients with breast cancer were recruited at the inpatient service of the Department of Breast Surgery of the Second Affiliated Hospital of Dalian Medical University and Liaoning Cancer Hospital from January 2010 and December 2014. Individual patients with breast cancer were diagnosed by pathological examination of two independent pathologists. Their blood samples were obtained for preparation of serum samples. Their surgical tumor samples were used for molecular classification, CSC and non-CSC isolation and miR-1 expression characterization. The demographic and clinical data of individual patients were obtained from medical records (Table 1). Written informed consent was obtained from individual patients, and the experimental protocol was approved by the Ethics Committee of Dalian Medical University.

**Table 1:** The characteristics of 45 enrolled cases

Various		*n*
Age	≤40 >40y	10 35
T stage	T1 T2	20 25
N stage	N0 N1	30 15
Clinical stge	DCIS IDC	15 30
Pathological stage	I II III	5 30 10
Molecular type	Luminal A Luminal B Her2 overexpression Basal	20 10 5 10

### Cell lines

293T cells and breast carcinoma cell lines, MCF-7 (lumina A, ER+, PR+, HER2-), MDA-MB-231 (basal-like, ER-, PR-, HER2-) and SKBR3 (ER-, PR-, Her-2+), were obtained from the American Type Culture Collection (ATCC, Manassas, USA) and cultured in 10% fetal bovine serum (FBS) DMEM medium (complete medium, Invitrogen, Grand island, USA) at 37°C in an atmosphere of 5% CO_2_.

### Isolation of breast CSC

ESA^+^CD44^+^CD24^−^lin^−^ CSCs and ESA^+^CD44^−^ CD24^+^lin^−^ non-CSC tumor cells were purified from six freshly surgical breast cancer tissues, as described previously [8]. Briefly, fresh breast tumor specimens were cut into small pieces and were digested with 1 mg/mL of collagenase type III (5 mL/g tissue, Worthington Biochemical, Lakewood, USA) in 5% fetal bovine serum (FBS) containing RPMI medium at 37°C for 2 h, and centrifuged. The cells were first stained with FITC-anti-CD2, APC-anti-CD3, PE-anti-CD10, FITC-anti-CD16, APC-anti-CD18, PEanti-CD31, and FITC-anti-CD326 lineage markers, as well as 7-AAD (BD Biosciences, San Jose, USA). Lineage+ and dead cells were first eliminated by flow cytometric sorting. Subsequently, the unstained lineage- cells were stained with PE-anti-CD24 and FITC-anti-CD44, and CD44+CD24− breast CSC and CD44-CD24+ non-CSC tumor cells were purified by flow sorting. Similarly, CD44^+^CD24^−^ cancer stemloid cells (CSCs) and CD44−CD24+ non-CSC cells were sorted from MCF 7 and SKBR3 cells. Briefly, MCF-7 and SKBR3 cells were stained with FITC-anti-CD44, APC-anti-CD24, and 7-aminoactinomycin D (7AAD). The 7AAD+ dead cells were eliminated and the CD44+CD24−, and CD44−CD24+ cells were sorted on a FACS vantage (FACSCALIBUR, BD Biosciences). The sorted CSCs and non-CSC cells had a purity of > 90% (Supplementary Figure S1). The purified CSCs were cultured in complete MammoCult™ Medium (10% human MammoCult™ Proliferation Supplements in MammoCult™ Basal Medium, Stem Cell Technologies, San Diego, USA) containing 50 ng/ml of leukemia inhibitory factor (LIF). The purified non-CSC tumor cells were cultured in complete medium.

### miRNA microarray

The profile of the Wnt/β-catenin signal-related miRNA expression in ESA+CD44+CD24−lin− breast CSCs and ESA+CD44−CD24+lin- non-CSC tumor cells was analyzed by miRNA microarray using Agilent human miRNA microarray kit (8 × 60 K, version16.0, Agilent Technologies, Santa Clara, USA), which contains capture probes for 1205 human miRNAs in the miRBASE database (release 16, available in http://www.mirbase.org). Briefly, the purified CSCs and non-CSC tumor cells were characterized for the expression of CD24 and CD44 by immunofluorescence and all of the purified CD44+CD24− CSCs and CD44−CD24+ non-CSC tumor cells had a purity of > 90%. Total small RNA was extracted from the CSC and non-CSC samples using mirVana™ miRNA Isolation Kit (Cat#AM1560, Invitrogen) and the quality of RNA samples was measured by the RNA integrity number (RIN) in an Agilent Bioanalyzer 2100 (Agilent Technologies). The miRNAs in each RNA sample were directly labeled with Cyanine 3-pCp and were hybridized to each slide (Cat#121, Thermo Shandon, Waltham, USA) using the miRNA Complete Labeling and Hyb Kit (Cat#5190–0456, Agilent Technologies), according to the manufacturer's instructions. After being washed, the slides were scanned and analyzed using the feature extraction software 10.7 (Agilent technologies). The raw data were first normalized to quantile algorithm, and further analyzed by the gene spring software 11.0 (Agilent technologies).

### Quantitative RT-PCR analysis of miR-1

Small RNA was extracted from MCF-7, SKBR3, MDA-MB-231, CD44+CD24− MCF-7/CSC, CD44− CD24+ MCF-7 non-CSC, SKBR3/CSC, SKBR3 non-CSC cells using the miRNeasy Mini Kit (#217004, Qiagen, Valencia, USA). The levels of miR-1 were determined by quantitative RT-PCR [12]. Briefly, individual RNA samples were digested with DNase I to remove the contaminated DNA, and the quality of RNA samples were determined by RIN. Subsequently, the RNA samples were reversely transcribed into cDNA and the relative levels of miR-1 to the control U6 RNA were determined by quantitative RT-PCR using the All-in one TM miRNA qRT-PCR reagent kit (#R0101L, GeneCopoeia, Rockville, USA). The data were analyzed by 2^−ΔΔCt^.

In addition, the relative levels of miR-1 to the control U6 RNA in MCF-7, SKBR3 cells, CD44+CD24− MCF-7/CSC or SKBR3/CSC that had been transfected with miR-1NC (5-UUCUCCGAACGUGUCACGUTT-3), miR-1inhibitor (5′-AUACAUACUUCUUUACAUUCCA-3′) and miR-1mimic (5-UGGAAUGUAAAGAAGUAUGUAU-3, Genechem, Shanghai, China), clinical surgical tumor tissues and serum samples were determined by quantitative RT-PCR.

### Dual luciferase report assay

The complementary 3′UTR sequences of the Wnt/β-catenin pathway-related mRNAs to which miR-1 potentially bound were searched from RefSeq. We found that miR-1 potentially bound to the 3′UTR of Frizzled 4, Frizzled 5, Frizzled 7, and TNKS2 (Supplementary Table S1). Accordingly, the DNA fragments for the 3′UTR of Frizzled 4, Frizzled 5, Frizzled 7, and TNKS2 were amplified from a cDNA library of breast cancer cells and cloned into the *Xho I* and *Sal I* sites near 3′ of the firefly luciferase gene in the dual-luciferase miRNA target expression vector, pmirGLO, respectively. The generated plasmids were sequenced. The impact of miR-1on the levels of luciferase activity was determined by dual-luciferase reporter assay using the dual-luciferase assay kit (DLR™, Promega, Madison, USA), according to the manufacturers' instruction. Briefly, 293T cells were cultured in 96-well plates overnight and transfected in triplicate with 0.5 μg individual plasmids that contained the 3′UTR of Frizzled 4, Frizzled 5, Frizzled 7, or TNKS2, together with either 0.75 μM miR-1NC or miR-1 mimic, for 48 h. The levels of dual-luciferase activity were measured [13].

CD44+CD24− MCF-7/CSCs and SKBR3/CSCs (1 × 10^5^ cells/well) were transfected in triplicate with 2 μM miR-1mimic, miR-1NC or miR-1inhibitor using lipofectamine (Life Technology) for 24 h. The cells (2 × 10^4^ cells/well) in each group were transfected in triplicate with TopFlash (20 ng) plasmid (#12456, Addgene, Cambridge, USA), Renilla luciferase thymidine kinase pRL-TK plasmid (5 ng, E2241, Promega) using lipofectamine for 48 h. The relative levels of β-catenin-dependent firefly luciferase activity in individual samples were determined by the Dual-Luciferase assay. Total value of reporter activity in each sample was normalized to Renilla luciferase activity.

### Flow cytometry analysis

MCF-7 and SKBR3 cells (1 × 10^5^ cells/well) were cultured in complete medium in 24-well plates overnight and transfected in duplicate with, or without, 2 μM miR-1 NC, miR-1 mimic or miR-1 inhibitor using lipofectamine (Life Technology) for 24 or 48 h. Some cells were used for quantitative analysis of the relative levels of miR-1 expression by quantitative RT-PCR. The remaining cells were stained with FITC-anti-CD44 and PE-anti-CD24 and the percentages of CD44+CD24− CSC cells in individual groups of cells were determined by flow cytometry.

### Western blot assay

CD44+CD24− MCF-7/CSCs and SKBR3/CSCs were transfected with, or without, miR-1NC or miR- 1mimic for 48 h. The relative levels of Frizzled 7, TNKS2 and c-Myc expression in MCF-7/miR-1NC. MCF-7/miR-1mimic, SKBR3/miR-1NC, SKBR3/miR-1mimic, untransfected MCF-7/CSC and SKBR3/CSC cells were determined by Western blot assays [10]. The primary antibodies included goat anti-Frizzled 7 (sc-31061), TNKS2 (SC-22854), mouse anti-c-Myc (sc-40) and rabbit anti-GAPDH (sc-25778, Santa Cruz Biotechnology, Santa Cruz, USA) and negative controls of rabbit or mouse IgG. The relative levels of each interesting protein to GAPDH were determined using the Gel pro4.0.

Similarly, the levels of Oct4 and Nanog as well as cytosol and nuclear β-catenin to control GAPDH or Lamin B1 (Santa Cruz Biotechnology) in MCF-7/CSC, MCF-7/miR-1NC, MCF-7/miR-1mimic, MCF-7/miR-1inhibitor, SKBR3/CSC, SKBR3/miR-1NC, SKBR3/miR-1mimic, SKBR3/miR-1inhibitor CSCs were characterized by Western blot using rabbit anti-Oct4 (sc-9081), anti-Nanog (sc-33759) and goat anti-Lamin B1 (sc-30264).

### Proliferation assay

The purified MCF-7/CSCs, SKBR3/CSCs, CD44− CD24+ non-CSC MCF-7 and SKBR3 cells were transfected with, or without, miR-1NC, miR-1 inhibitor, miR-1 mimic for 48 hours, respectively. The generated MCF-7/miR-1NC, MCF-7/miR-1inhibitor, SKBR3/miR-1NC, SKBR3/miR-1mimic and untransfected control MCF-7 and SKBR3 CSCs (1 × 10^5^ cells/well) were cultured in triplicate in 96-well plates for 24 or 48 h. Similarly, the generated CD44− CD24+ MCF-7, MCF-7/miR-1NC, MCF-7/miR-1 inhibitor, SKBR3, SKBR3/miR-1NC and SKBR3/miR-1mimic non-CSC cells were cultured for 24 or 48 hours. During the last 4-h incubation, the cells were treated with MTT. The resulting formazan was dissolved in DMSO and measured for the absorbance at 540 nm.

### Wound healing assay

The migration of MCF-7/miR-1NC, MCF-7/miR-1inhibitor, SKBR3/miR-1NC, SKBR3/miR-1mimic and control breast CSCs was determined by wound healing assay [14]. Briefly, individual types of cells (5 × 10^5^ cells/well) were cultured in triplicate in 24-well plates and when they reached nearly 90% confluence, the cells were scratched with tip across the wells. The cells were continually cultured for 24 or 48 h and imagined. The migration distance of cells was measured using ImageJ software (NIH, Rockvill***e***, USA).

### Transwell migration assay

The migration of breast CSCs was determined by transwell migration assay [3]. Briefly, MCF-7/miR-1NC, MCF-7/miR-1inhibitor, SKBR3/miR-1NC, SKBR3/miR-1mimic and control breast CSCs (1 × 10^5^ cells/well) were cultured in triplicate in the top chambers of 24-well transwell plates (8.0 mm-pore, Corning) and complete MammoCult™ Medium were added into the bottom chambers. After 24- or 48-h culture, the cells on the surface of the top chamber membrane were removed with a cotton swab. The migrated cells on the bottom surface of the top chamber membranes were fixed with 95% alcohol, stained with hematoxylin solution, and counted in 10 random fields under a microscope (magnification × 200).

### Xenograft breast tumor model

The experimental protocol was approved by the Animal Research and Care Committee of Dalian Medial University. CD44+CD24− MCF-7/CSCs were sorted by flow cytometry and transfected with pGPU6/GFP/miR-1NC, pGPU6/GFP/miR-1mimic or pGPU6/GFP/miR-1 inhibitor (GenePharma, Shanghai, China). The cells were treated with G418 for three weeks to generate MCF-7/miR-1NC, MCF-7/miR-1mimic or MCF-7/miR-1inhibitor CSCs. The levels of miR-1 expression in individual types of cells were determined by quantitatively RT-PCR. Female athymic nude mice at 6–8 weeks of age were from Silaike Laboratory Animal (Shanghai, China) and housed in a specific pathogen free facility with free access to autoclaved water and food. Individual mice were randomized and injected with 5 × 10^5^ MCF-7/miR-1NC, MCF-7/miR-1mimic or MCF-7/miR-1inhibitor CSCs (0.1 ml in FBS-free medium) into their left fat pad (*n* = 8 per group). The growth of implanted tumors was monitored and the volumes of tumors were calculated [15]. At the end of the experiment, the relative levels of miR-1 expression in individual groups of tumors were tested by quantitatively RT-PCR.

### Statistical analysis

Data are expressed as the means ± SD or median and range. The difference among groups was determined by ANOVA and post hoc Fisher's least significant difference using SPSS software, version 16.0. A *P*-value of < 0.05 was considered statistically significant.

## SUPPLEMENTARY FIGURES AND TABLES


