# Supplementary material for: MicroRNA-1 down-regulates proliferation and migration of breast cancer stem cells by inhibiting the Wnt/β-catenin pathway
Source: Oncotarget. 2015 Oct 19;6(39):41638–49. doi: 10.18632/oncotarget.5873 (PMC4747178; doi:10.18632/oncotarget.5873)
Supplement: Supplementary file 1 [file oncotarget-06-41638-s001.pdf]

SUPPLEMENTARY FIGURES AND TABLES

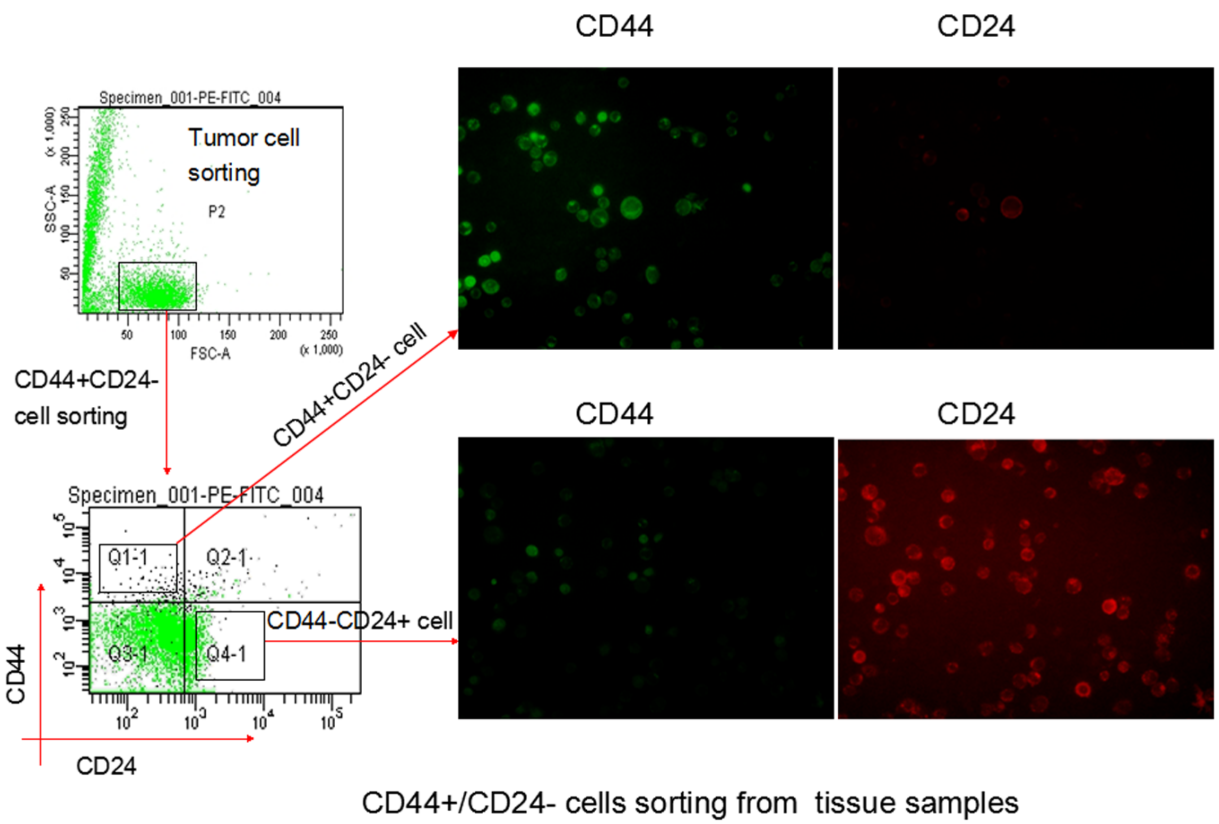

Supplementary Figure S1: Flow cytometry sorting CD44+CD24− breast CSCs and CD44− CD24+ non-CSC tumor cells from freshly dissected breast cancer specimens. $ESA^{+}lineage^{-}$ tumor cells from six freshly surgical breast cancer specimens were first selected and stained with 7AAD, APC-anti-CD24 and FITC-anti-CD44, followed by flow cytometry sorting. The live cells were gated and CD44+CD24− CSCs and CD44− CD24+ non-CSC tumor cells were sorted. Subsequently, the sorted cells were examined under a fluorescent microscope. Data are representative FACS charts and images.

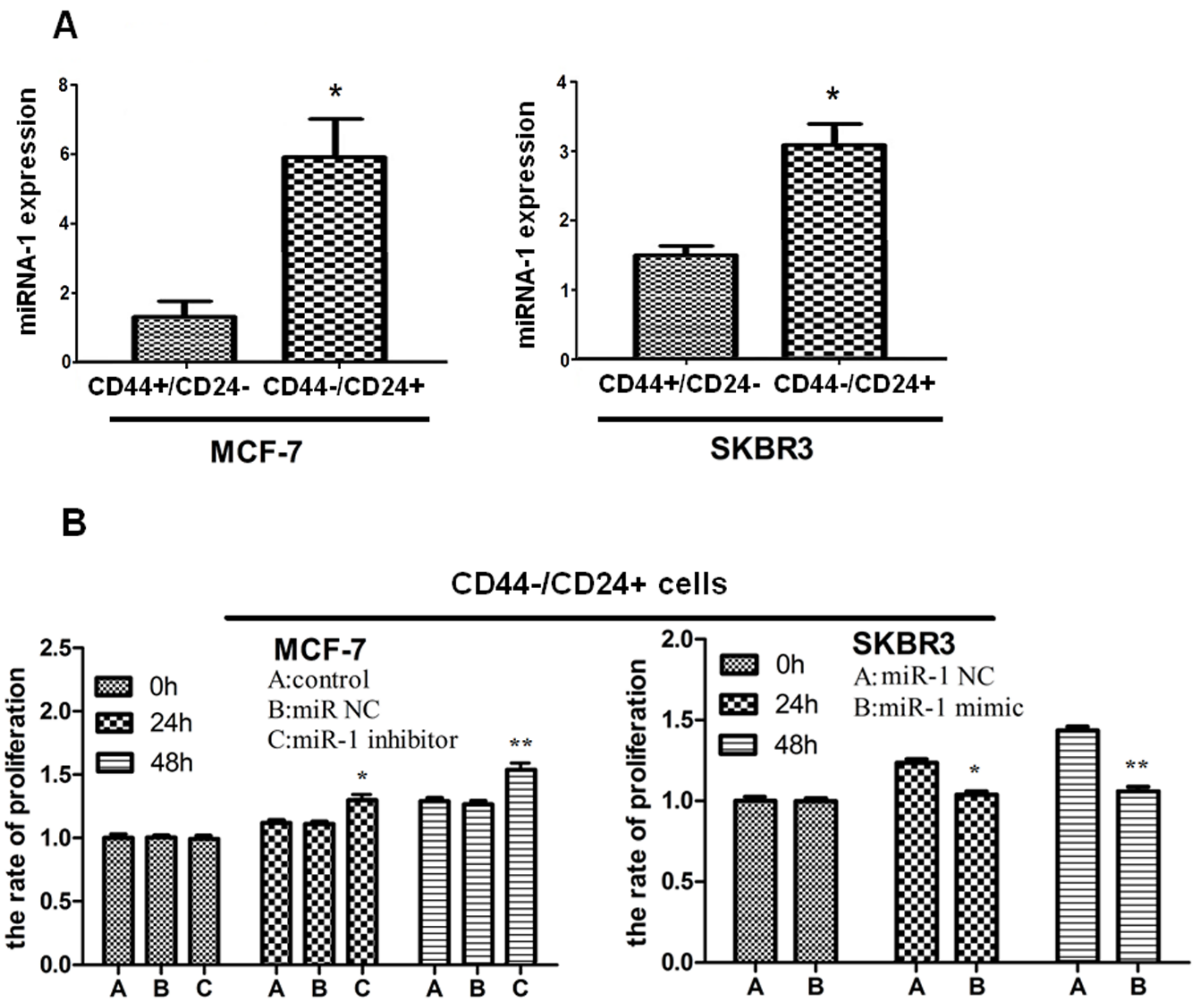

Supplementary Figure S2: The expression and function of miR-1 in CD44-CD24+ non-CSC tumor cells. CD44+CD24− CSCs and CD44− CD24+ non-CSC tumor cells were sorted from MCF-7 and SKBR3 cells. Their miR-1 expression was determined by quantitative RT-PCR **A.** The CD44−CD24+ non-CSC tumor cells were transfected with, or without, miR-1NC, miR-1inhibitor or miR-1mimic for 24–48 hours. The proliferation rates of different groups of cells were determined by MTT **B.** Data are expressed as the mean ± SD of each group of cells from three separate experiments. The proliferation rates in the untransfected SKBR3 cells were similar to that of SKBR3/miR-1NC and designated as 1. $*p < 0.05$, $**p < 0.01$ vs. the controls.

Supplementary Table S1: The potential sequences in the 3'UTR of Frizzled 4, 5, 7 and TNKS2 for miR-1 binding

	Predicted sequences (top) for miR-1 (bottom) binding
Position 996-1002 of 3’UTR of Frizzled 4	5′…UAGGCCUCUCCUUUC---ACAUUCCA…
miR-1	3′ UAUGUAUGAAGAAAUGUAAGGU
Position 996-1002 of 3′UTR of Frizzled 7	5′…AUUUGGUUGAGAUAAACAUUCCU…
miR-1	3′ UAUGUAUGAAGAAAUGUAAGGU
Position 220-226 of 3′UTR of TNKS2	5′…CACUUUAACAGAUGCCAUUCCAG…
miR-1	3′ UAUGUAUGAAGAAAUGUAAGGU
Position 2232-2239 of 3′UTR of TNKS2	5′…UACUUGUACUGUAUCACAUUCCA..
miR-1	3′ UAUGUAUGAAGAAAUGUAAGGU
Position 713-720 of 3′UTR of TNKS2	5′…UUGUAUGUAUGAGACACAUUCCA…
miR-1	3′ UAUGUAUGAAGAAAUGUAAGGU

Supplementary Table S2: The differentially expressed miRNAs potentially involved in the Wnt/β-catenin signaling in breast CSCs

up-regulated miRNAs	down-regulated miRNAs
miR-302d	miR-671-5p
miR-148b	miR-7d
miR-16-2	miR-1
miR-367	miR-584
miR-302b	miR-499-5p
miR-487a	miR-26a
miR-574-5p	miR-197
miR-1826	miR-1225-5p
miR-1246	miR-146a
miR-29a	miR-224
miR-181b	miR-135b
miR-140-5p	miR-154
miR-135a	miR-29a
miR-1280	
